# MRI-based navigated cryosurgery of extra-abdominal desmoid tumors using skin fiducial markers: a case series of 15 cases

**DOI:** 10.1186/s12891-023-07074-6

**Published:** 2023-12-15

**Authors:** Ortal Segal, Amit Benady, Eliana Pickholz, Joshua E. Ovadia, Ido Druckmann, Solomon Dadia, Ehud Rath, Assaf Albagli, Ben Efrima

**Affiliations:** 1 National Department of Orthopedic Oncology, Tel Aviv Medical Center, Tel Aviv, Israel; 2 Division of Orthopaedic Surgery, Tel Aviv Medical Center, Tel Aviv, Israel; 3 Sackler Faculty of Medicine, Tel Aviv University, Tel Aviv, Israel; 4 Division of Radiology, Tel Aviv Medical Center, Tel Aviv, Israel; 5 Levin Center for Surgical Innovation and 3D printing, Tel Aviv Medical Center, Tel Aviv, Israel

**Keywords:** MRI-based navigation, Fiducial markers, Percutaneous CRA, Desmoid Tumor, 3D planning

## Abstract

**Background:**

Precision surgery is becoming increasingly important in the field of Orthopaedic Oncology. Image-guided percutaneous cryosurgery (CRA) has emerged as a valid treatment modality for extra-abdominal desmoid tumors (EDTs). To date, most CRA procedures use CT-based guidance which fails to properly characterize tumor segments. Computer-guided MRI navigation can address this issue however, the lack of a fixed landmark for registration remains a challenge. Successful CRA correlates directly with precision approaches facilitated by intraoperative imaging guidance. This is the first study that attempts to assess the feasibility and efficacy of a novel approach of using skin fiducial markers to overcome the challenge of a MRI-based navigation CRA for symptomatic or progressive EDTs.

**Methods:**

In this retrospective study conducted between 2018 and 2020, 11 patients at a single center with symptomatic or progressive EDTs were treated with CRA using intraoperative MRI navigation. Fifteen cryosurgery procedures were performed, each adhering to a personalized pre-operative plan. Total tumor size, viable and non-viable portions pre- and post-operation, and SF-36 questionnaire evaluating subjective health were recorded.

**Results:**

All CRAs demonstrated 100% adherence to the predetermined plan. Overall, tumor size decreased Median= -56.9% [-25.6, -72.4]) with a reduction in viable tissue, (Median= -80.4% [-53.3, -95.2]). Four patients required additional CRAs. Only one patient’s tumor did not reduce in size. One patient suffered from local muscle necrosis. Pre-operation, the average physical and mental scores 41.6 [29.4, 43] and 26.3 [17.6, 40.9] respectively. Post-operation, the average physical and mental scores were 53.4[38, 59.7] and 38 [31.2, 52.7] respectively.

**Conclusion:**

These findings provide an early indication of the feasibility and efficacy of performing percutaneous cryosurgery using skin fiducial marker registration for MRI-computed navigation to treat EDTs safely. Larger cohorts and multicenter evaluations are needed to determine the efficacy of this technique.

## Background

Desmoid tumors (DTs), also known as aggressive fibromatosis, are rare aggressive benign tumors that arise from clonal proliferation of spindle cells. With an incidence rate of roughly 2–4 cases per million in the general population, DTs account for 0.3%-0.1% of all solid tumors [1]. DTs occur in both sporadic and hereditary forms. Sporadic DTs account for 85-95% of cases and give rise to extra-abdominal desmoid tumors (EDTs), while the rare familial DTs account for 5–15% of cases and are associated with familial adenomatosis polyposis syndrome (FAP) [2].

Although EDTs have been shown not to metastasize, they often behave aggressively in their local environments and are thus associated with high morbidity [3, 4]. The gold standard of treatment previously included either en-block resection, radiotherapy, or systemic chemotherapy. However, these methods are associated with a high frequency of local recurrence and complications [5, 6]. Consequently, the treatment paradigm has shifted towards a “wait and see” policy of “active surveillance” [7–9]. This approach, however, cannot be applied in all cases. As 36% of tumors grow and progress, they often cause debilitating symptoms with the potential to disable patients or jeopardize the function of adjacent critical organs [10]. To improve available treatment options, new modalities have emerged and are currently being implemented for treatment of EDT. Percutaneous cryosurgery (CRA) as an alternative has shown to reduce tumor burden and improve symptoms. CRA also maintains a low incidence of morbidity. As a result, many authors have advocated to establish CRA as a valid treatment option.

Successful CRA procedures to date correlate directly with the utilization of precise intraoperative image guidance, mainly CT-based [11–13]. An important limitation of this imaging modality is its ability to distinguish between tumor and healthy tissue. This does not allow for tumor segmentation of viable and non-viable tissue in a precise manner which an MRI scan is capable of.

Computerized navigation is currently used to guide precision surgeries in various fields of orthopedic surgery. It allows for optimal intraoperative execution of preoperative planning and reduces the risk of damaging untargeted structures during surgery [14–16]. MRI-computed navigation depends on a critical procedure called registration. During registration, the surgeon identifies a predetermined landmark on the patient and matches it to preoperative images, thus enabling projection of these images onto the navigation system. Historically, this process relied on fixed bony landmarks as a reference point, since elastic soft tissue was unreliable. Consequently, MRI computer navigation could not be used for soft tissue tumors lacking exposed bony landmarks [17].

Adhesive skin fiducial markers can combat this challenge, as they are easy to apply and identify on advanced imaging like MRI. This method is commonly carried out in fields such as diagnostic radiology, interventional radiology, and neurosurgery [18–20]. In this study, we proposed a novel 3 steps technique to improve the accuracy and safety of cryosurgery: preoperative computed 3D-planning with skin fiducial markers for image, MRI-based intra-operative navigation, and imaging-based intraoperative validation of cryo-needle placement. The purpose of this study was to evaluate the feasibility and efficacy of fiducial-based MRI-navigated cryosurgery as a treatment for symptomatic or progressive EDT.

## Materials and methods

### Patient selection

This retrospective study occurred between 2018 and 2020 in a single center and received Institutional Review Board approval. Inclusion criteria called for patients 18 years or older presenting with either a progressively growing tumor, demonstrated by two sequential MRI scans taken 3 months apart, or with a highly symptomatic tumor that was unresponsive to conventional analgesic treatment. All patients in this study were reviewed by the institutional multi-disciplinary tumor board, comprised of an oncologist, radiologist, pathologist, and orthopedic oncologist. All treatment options were considered prior to transitioning from a hands-off, wait-and-see policy to cryosurgery.

### Preoperative planning protocol

#### 3D module reconstruction

**Fig. 1:**
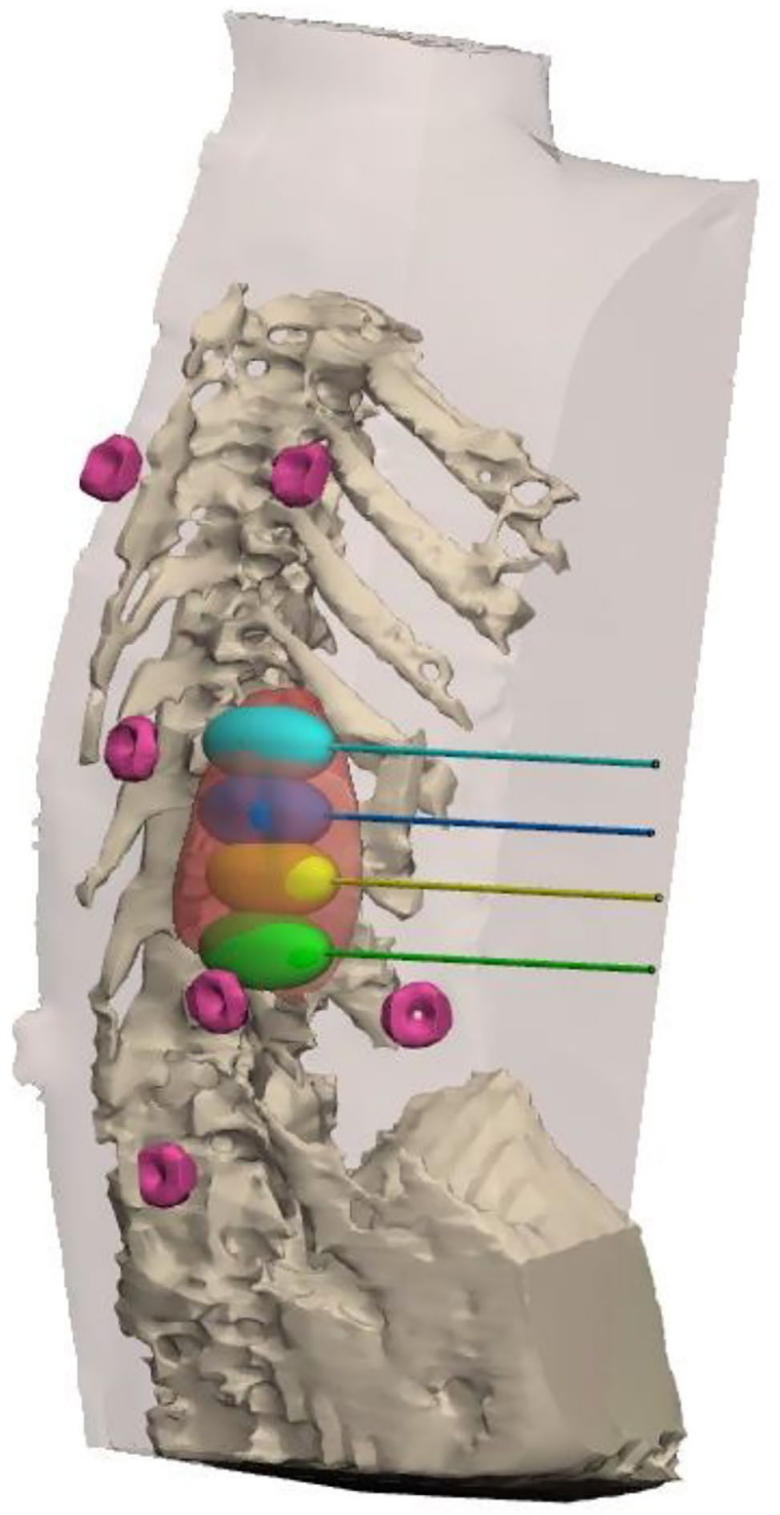
3-Dimmensional digital planning of the CRA procedure

An MRI scan was used to generate a computerized three-dimensional module. The module was generated using Mimics® software (v23, Materialise, N.V. Leuven, Belgium). A customized preoperative plan was constructed for each individual patient (Fig. 1). The module also considered potential at-risk structures such as major vessels, nerves, and vital organs, to ensure that any potential plan would not jeopardize them. To achieve optimal tumor coverage, the number of needles, trajectory, and placement were planned based on the generated modules. An ice ball with a radius of 4 cm was generated at the end of each cryo-needle to use as a reference for the ablation zone of each needle. The total ablation zone was determined to equal the sum of all the individual ice balls.

#### Pre-operative MRI and fiducial markers placement

**Fig. 2:**
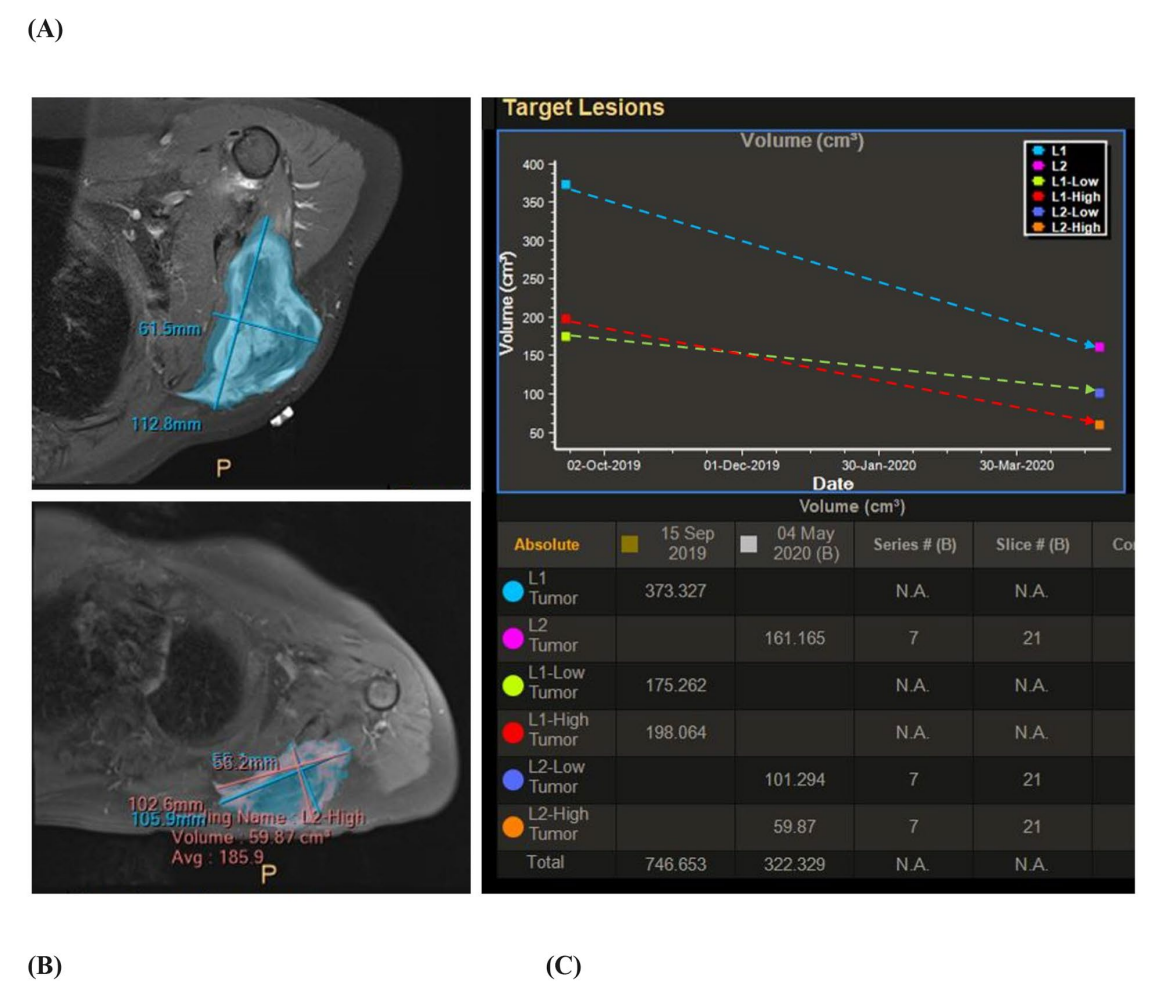
Gaussian Mixture Model (GMM) segmentation of a desmoid tumor. (**A**) Tumor before CRA. (**B**) Tumor after CRA. Both in A and B note the difference between the high and low intensity (sub-segmentation), which represent the viable and non-viable tissue, respectively. (**C**) Light blue- total tumor size before ablation. Pink- total tumor size after ablation. Red- high intensity (i.e., viable tissue) before ablation. Orange- high intensity after ablation. Green- low intensity (i.e., necrotic tissue) before ablation. Purple- low intensity after ablation. Light blue, red and green dashed lines show the reduction in total size, viable and non-viable tissue, respectively

One day prior to ablation, skin fiducial markers (pinpoint, beekly medical LTD) were scattered in a random pattern around the tumor and a preoperative MRI scan was performed. The markers were kept in place for surgery the following day to ensure that the intraoperative registration included the precise location of the markers displayed in the navigation system. In addition, the MRI scans were used to re-evaluate the tumor size, location, and composition in order to assess for any deviations from the preoperative plan (Fig. 2).

### Intraoperative protocol

#### Preparation and image registration

**Fig. 3:**
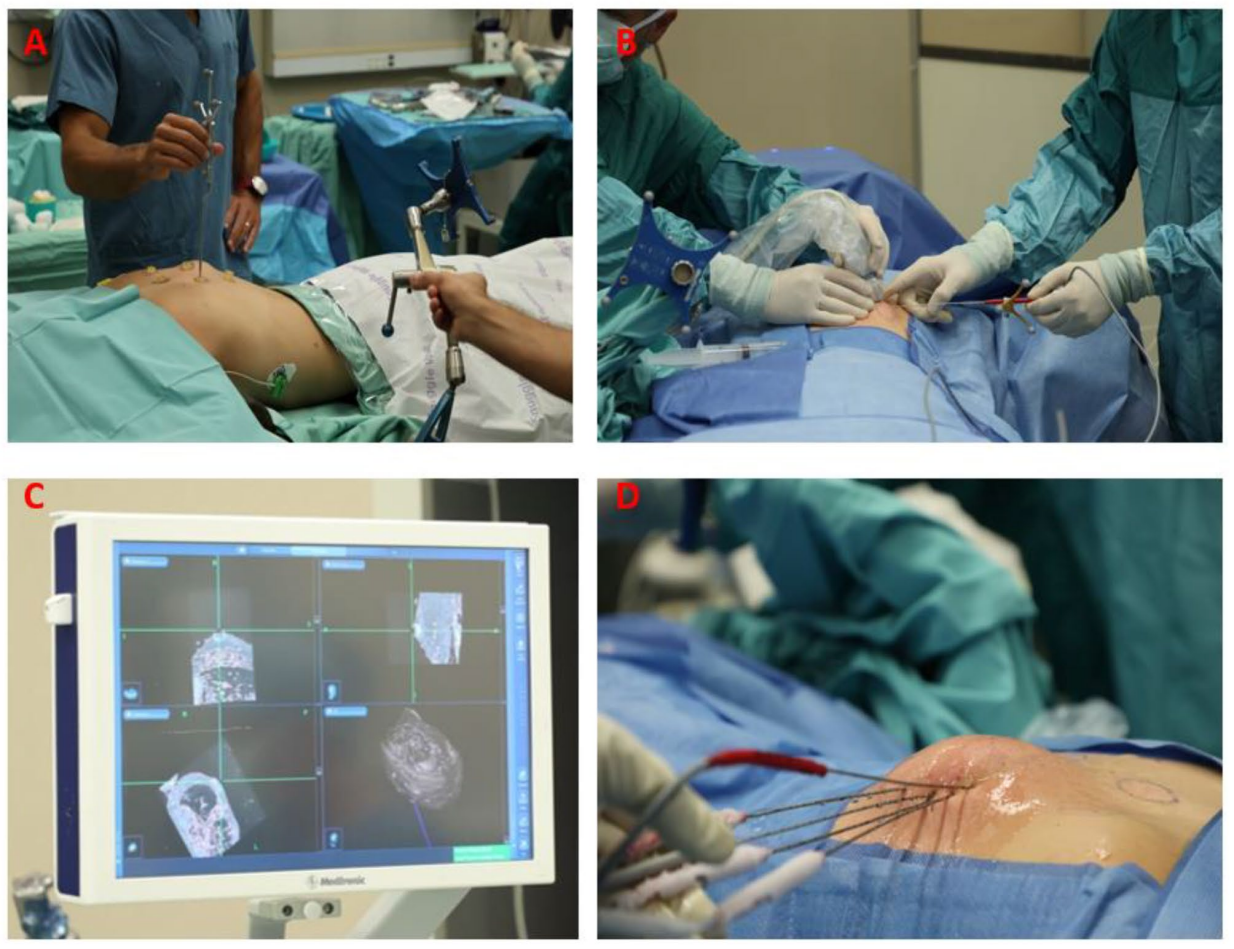
**(A)** Pointer registration of each fiducial marker. **(B)** The sure track system is attached to cryo-needle and calibrated. The needle can be inserted into the tumor using navigation according to pre-operative 3D planning. Ultrasonography was used to verify position. **(C)** Stealth station MRI screen navigation shows bright and dark areas, indicating desmoid tumor viability. **(D)** Cryo-needles inserted according to the preoperative plan, using warm saline to protect the skin. A thermometer guide needle was also used to monitor and maintain core tumor temperature of below − 40 degrees Celsius

All interventions were performed by first author (O.S) a fellowship-trained orthopedic oncologist. Surgeries were performed in an operating room on patients placed under general anesthesia. Stealth-station navigation system (StealthStation®, Medtronic Sofamor, Danek) was set to cranial model in order to allow for optimal soft tissue demonstration. Then, the MRI scans taken the previous day were uploaded to the stealth station as DICOM files. Registration was performed for each individual skin fiducial marker using pointer (medtronics). At the end of the registration and calibration process, the skin fiducial markers were then removed. Each individual cryo-needle was calibrated to the generated image using the sure-tract system (Danek and Sure-Track system by Medtronic). Using MRI based navigation, the cryo-needles (Galil medical-ice rod) were placed inside the tumor mass according to the pre-operative plan. The location and position of each of the needles were confirmed using an ultrasound (US) performed by author I.D, a fellowship trained radiologist. After placing all cryo-needles, cone beam CT scan was performed as a cautionary measure to verify that the chosen needle location was identical to the preoperative model (O-arm scanner Medtronic Sofamor; Dannek). The ablation protocol subsequently began (Fig. 3).

### Cryosurgery protocol

The treatment protocol included ten minutes of freezing followed by five minutes of thawing after which, an additional ten minutes of freezing. Neuromonitoring was precautionarily used in procedures that were performed in proximity to major nerves. Skin temperature was closely monitored to avoid any thermal damage. To protect the underlying skin, a barrier was created by continuously injecting warm normal saline into the subcutaneous layer to hydro-dissect the skin from the underlying tissue. All procedures were performed on an ambulatory basis. Patients were discharged the same day of operation without the need for any dressings.

### Postsurgical surgical image analysis

Patients underwent MRI scanning at 3-, 6-, and 12-months post-cryosurgery. The IntelliSpace Discovery software platform (Philips Healthcare) which employed a novel Gaussian mixture model (GMM) algorithm developed specifically for post-surgical imaging analysis was then utilized. This method employed a semi-automatic 3D segmentation tool to successfully identify variations in complex tissues at the macroscopic level enabling volumetric segmentation of the desmoid tumor tissue of each patient (Fig. 3) [21]. Images were compared pre- and post-cryosurgery. Total Tumor Volume (TTV) was evaluated and compared to the pre-operative assessment. Tumors were then further categorized into viable (VTV) and highnon-viable tumor volume (NVTV) and were evaluated semi-automatically using Phillips software according to their density.

Tumor dimension and composition were assessed using the patients’ series of consecutive postoperative MRI scans (3, 6, and 12 months). Minimum follow-up time was 12 months. Treatments which yielded a decline in viable tumor volume were deemed successful.

### Data collection and follow up

Medical records were reviewed for demographic and clinical data, tumor volume, tumor location, and symptoms prior to the intervention. Additionally, prior treatments were reviewed and documented. Intra-operative CT scans were used to compare each element of the procedure with the pre-operative 3D model, including the number of needles used and potential involvement of untargeted structures. Any discrepancy between the pre-operative plan and final execution was documented. Patient follow-up was conducted in the outpatient clinic at 3, 6, and 12 months. Pre-operative and post-operative symptoms were compared. The 36-item Short Form Health Survey (SF-36) was administered pre- and 12 months post-operation to evaluate individual patient health status and compare disease burden.

### Statistical analysis

Continuous data were assessed for normal distribution using Shapiro-Wilks test. Since most of the continuous data was not normally distributed the data was presented as Median + interquartile range (IQR). Comparisons were conducted using IBM SPSS software (V26).

## Results

Between 2018 and 2020, eleven patients (39.9 ± 15.6 years of age; 6 females, 5 males) with symptomatic EDTs were treated with MRI navigated cryosurgery. Tumor locations are described in Table 1. Four patients had previous treatments prior to the CRA (Table 1).

**Table 1:** Demographic patient data, tumor locations, comorbidities

Patient	Age	Gender	Ethnicity	Location	Previous Tx	Patient comorbidities
1	46	M	Caucasian	Posterior chest wall	N/A	HTN
2	33	M	Caucasian	Scapula	Chemotherapy	N/A
3	46	F	Caucasian	Thigh	N/A	N/A
4	18	F	Caucasian	Foot	Chemotherapy, Biologic Tx, Resection	N/A
5	65	M	Caucasian	Shoulder	N/A	HTN, DM, COPD, NAFLD
6	22	M	Caucasian	Shoulder	N/A	N/A
7	42	F	Caucasian	Sacrum	N/A	N/A
8	55	F	Caucasian	Shoulder	Resection	N/A
9	57	M	Caucasian	Thigh	N/A	N/A
10	28	F	Caucasian	Lower back	N/A	N/A
11	27	F	Caucasian	Axilla	Chemotherapy	N/A

CRA = Cryosurgery

Tx = Treatment

### 3.1 Surgical procedure

Each of the total 15 cryosurgery procedures followed a personalized pre-operative plan. Each procedure included 2–12 needles, and all needles demonstrated 100% fit to the pre-determined plan. Eight patients (73%) required only a single procedure. Three patients (27%) demonstrated additional tumor growth, required additional cryosurgery procedures, and eventually showed improvement, (two patients required two procedures and one patient three procedures). The median surgical time was 82 min [IQR 65 90]. According to SIR adverse event classification guidelines, only one participant had a mild complication of muscle necrosis (grade 1) [22]. (Table 2).

**Table 2:** Procedural operative data and immediate post-operative complications

Patient	# of CRA procedures	Average surgical time (min)	# of cryoneedles in pre-op plan	# of needles used	Complications
1	2	**77**	11	11	N/A
2	2	**110**	12	12	Muscle necrosis
3	3	**82**	12	12	N/A
4	1	**90**	11	11	N/A
5	1	**110**	12	12	N/A
6	1	**89**	12	12	N/A
7	1	**69**	6	6	N/A
8	1	**89**	2	2	N/A
9	1	**65**	8	8	N/A
10	1	**65**	5	5	N/A
11	1	**45**	7	7	N/A

### Post-operative analysis

Overall, total tumor volume (TTV) decreased from pre- to post-operation. Median tumor volumes pre- and post-operation were 158.5 mL [32.9, 331.7] and 77.9 mL [10.4, 201.5], respectively, yielding a reduction of 56.9% [25.6%, 72.1%]. Only one patient’s tumor did not reduce in size. Before the operation, tumors consisted of 43.3% [21.5%, 51.0%] non-viable tissue and 56.7% [48.9%, 78.5%] viable tissue, compared with 60.7% [54.6%, 79.9%] non-viable tissue and 37.5% [11.8%, 43.1%] viable tissue following surgery. An increase in the percentage of non-viable tissue volume (NVTV) from the whole tumor was observed (median= -22.8% [6.6%, -83.3%], while a reduction of 68.6% [-38.8 -175.7] was observed in percentage of viable tissue volume (VTV). (Tables 3 and 4).

**Table 3:** Imaging results pre-and post-cryosurgery

Patient	Location	Total volume pre-op	Non-viable tumor volume pre-op	Viable tumor volume pre-op	Total volume post-op	Non-viable tumor volume post-op	Viable tumor post-op
1	back	392.7	225.2	167.5	162.6	84.4	78.2
2	back	615.2	132.1	483.1	471.4	376.8	94.6
3	thigh	158.5	32.6	125.9	201.5	153.9	47.6
4	foot	38.4	15.4	23.0	10.7	6.5	4.2
5	shoulder	331.7	146.2	185.5	246.8	147.1	99.7
6	shoulder	196.3	84.9	111.4	44.8	39.5	5.3
7	back	115.0	72.4	42.6	77.9	77.6	0.3
8	shoulder	15.1	6.6	8.5	6.5	3.7	2.8
9	thigh	168.7	86.1	82.6	99.2	54.2	45
10	back	32.9	7.9	25.0	10.4	6.5	3.9
11	Arm	2.3	0	2.3	0	0	0

**Table 4:** Descriptive statistics

Patient	Median	25th Percentile	75th Percentile	95% Lower CL for Median	95% Upper CL for Median
Total volume pre	158.5	32.9	331.7	32.9	331.7
Non-viable volume pre	72.40	7.90	132.10	7.90	132.10
Viable volume pre	82.6	23.0	167.5	23.0	167.5
Total volume post	77.90	10.40	201.50	10.40	201.50
Non-viable volume post	54.2	6.5	147.1	6.5	147.1
Viable volume post	5.3	2.8	78.2	2.8	78.2
Δ % total volume	-56.95	-72.14	-25.60	-72.14	-25.60
% Total Non-viable volume pre	43.25	21.47	51.04	21.47	51.04
% Total Viable volume pre	56.75	48.96	78.53	48.96	78.53
% Total Non-viable volume post	60.75	54.64	79.93	54.64	79.93
% Total Viable volume post	37.50	11.83	43.08	11.83	43.08
Δ % Viable volume	-68.66	-175.70	-38.86	-175.70	-38.86
Δ % Non-viable volume	-22.81	-83.18	6.68	-83.18	6.68

*CL – Confidence Limit

### Subjective health questionnaire

To evaluate subjective health, SF-36 questionnaires pre- and post-operation were administered. Pre-operation, the average physical and mental scores were 41.6 [29.4, 43] and 26.3 [17.6, 40.9] respectively. Post-operation, the average physical and mental scores were 53.4 [38, 59.7] and 38 [31.2, 52.7] respectively.

## Discussion

This study demonstrates the feasibility, efficacy, and safety of fiducial-based MRI navigated cryosurgery for EDTs. Imaging-based preoperative planning with the use of skin fiducial markers, intraoperative MRI-computed navigation, and imaging-based confirmation of cryo‐needle placement are precise steps that can improve the overall efficacy of cryosurgery. Our preoperative plan was able to accurately predict the intraoperative of needle placement and the reductions in both Total Tumor Volume (TTV) and Viable Tumor Volume (VTV) were achieved while maintaining a low post-operative complication rate. SF36 scores demonstrated improvements in both mental and physical health scores.

Desmoid tumors are associated with high rates of morbidity and local recurrence [23]. Complete removal is the preferred outcome for most tumor surgeries, in the case of desmoids however, the end goal of any treatment should aim to arrest or reduce tumor progression and alleviate symptoms to mitigate morbidity. Once a period of watchful-waiting has failed, the current guidelines set forth by the National Comprehensive Cancer Network call for chemotherapy, radiotherapy, and en‐bloc surgical resection as primary therapy [8]. A recent multicentral multinational study found that among patients treated by surgical resection, 36.6% had local recurrence and 63.4% showed stable disease. Alternatively, patients treated with chemotherapy, radiotherapy or hormonotherapy had 40.2% experienced recurrence and 59.8% had a stable disease (defined as no evidence of disease or stable disease) [4]. Furthermore, in a review of 22 articles, Nuyttens et al. [24], reported extensively on complications. They described combined rates for mild and moderate complications in 27% and 45% of cases for surgery and radiation, respectively. It was further noted that in the surgically treated cohort, severe complications such as amputation or major disability were reported in up to 4% of cases.

Cryosurgery as an alternative treatment option has gained immense popularity in the last decade as a relatively effective and safe alternative to the current guidelines. A recent systematic review and meta-analysis found that out of the proportion of non-progressive disease rates (85.8%), major and minor complications rate ranged from 2.4 to 14.2% and 4.8–23.3%, respectively, for all procedures [25]. The success rate of percutaneous cryosurgery directly correlates with the accuracy and precision of the ablation process [12, 26]. MRI is the imaging modality of choice for optimal visualization of EDTs at preoperative planning and post-operative follow up [27]. Therefore, MRI-computed navigation should simplify and improve cryosurgery once accurate image registration is achieved.

Several findings from this study provide an early indication of the advantages of this ablation MRI-navigated cryosurgery protocol. Firstly, by using an 3DM and carefully plan the needles trajectory and placement we were able to predict the number of cryoneedles required to cover the tumor mass. the similarities between needle placement and number during the surgery compared with the preoperative 3D model indicate that the preoperative plan can be implemented using this navigation protocol. This finding could be attributed to the fact that the preoperative and intraoperative images are both MRI-based scans of the same fiducially marked location and are, therefore, identical to one another. Additionally, obtaining a necessary scan that includes the fiducial marker placement requires MRI scanning one day prior to the operation. In turn, this allows the surgeon to assess the tumor for any changes in size or composition. The surgeon can then plan any adjustments to the preoperative plan.

In the current cohort, a notable reduction was observed in the TTV and VTV in all but one patient. This cohort demonstrated a reduction of 53.7% ± 33.8% and 31% ± 16.1% for TTV and VTV, respectively. This study reinforces findings from previous studies that similarly demonstrated significant reductions in both total and viable tumor volume after percutaneous cryosurgery [4, 11, 26, 28]. Notably, one patient showed a reduction in VTV but not TTV. This patient was offered a revision cryosurgery but sought alternative treatment options instead. Four patients experienced local recurrence and subsequently underwent revision cryosurgeries which, importantly demonstrates that CRA could be performed repeatedly with low morbidity.

A total of 15 cryosurgeries were performed in 11 patients using the current navigation precision protocol. Only one participant (9%) experienced a post-operative complication of skin necrosis classified as a mild complication (grade 1) according to SIR adverse event guidelines [22]. This cohort had significantly lower complication rates compared to cryosurgery and both other modalities. A similar study by Auloge et al. [29] reported a complication rate of 36.6%, consisting mostly of mild complications. Furthermore, Tremblay et al. [12] reported complication rates of 6.7% and 13.3% for major and minor complications, respectively. In this study, the preoperative plan and intraoperative execution successfully achieved a low rate of postoperative complications. These outcomes further validate the efficacy of the current navigation precision protocol.

SF-36 questionnaires were used to evaluate the individual functional status of each patient by comparing their 12 months follow-up score to their baseline pre-operative values. An increase in physical and mental statuses were found in our cohort. Overall, eight participants showed improved physical and mental status, while one participant had an increased mental status but decreased physical status, and two participants had increased physical but decreased mental status. Overall, this data further reinforces the promising clinical results by demonstrating improved patient-reported outcomes.

Application of this study extends beyond merely establishing a new CRA technique. Although MRI navigation has proven to be a valuable tool for precision surgery, its application to soft tissue tumors of the trunk and extremities has been limited by difficulties in the registration process. In a previous study, Eccles et al. [17] successfully achieved margin-free resection with remarkable accuracy while using skin fiducial marker-based MRI navigation to resect soft tissue tumors from cadavers. However, to the best of our knowledge, the current study represents the first attempt to apply this navigation technique in vivo. This study’s success indicates the potential to use this technique for future cryosurgery as well other surgical approaches. Introducing a new surgical technique should follow a specific, stepwise process including: (1) concept/theory formation, (2) procedure development and exploration, (3) procedure assessment, and (4) long-term, ideally evidence-based studies [30]. We believe this study contributes significantly to the second stage.

This study was not without limitations. Importantly, it was a retrospective study based on a small study population. The rare incidence of this tumor makes it difficult to aggregate a large volume of patients. We recognize the need to expand this study and test this procedure on a larger sample size. A long-term follow-up is necessary to validate this procedure.

## Conclusion

These findings provide an early indication of the feasibility and efficacy of performing percutaneous cryosurgery using skin fiducial marker registration for MRI-computed navigation to treat EDTs safely. Larger cohorts and multicenter evaluations are needed to determine the efficacy of this technique.

## Data Availability

The datasets used and/or analyzed during the current study available from the corresponding author on reasonable request.

## List of abbreviations

CRA: cryosurgery
EDT: extra-abdominal Desmond tumors
FAP: familial adenomatosis polyposis syndrome
GMM: Gaussian Mixture Model
TTV: total tumor volume
VTV: viable tumor volume
SD: standard deviation

## References

[1] Sakorafas GH, Nissotakis C, Peros G (2007). Abdominal desmoid tumors. Surg Oncol.

[2] Lewis JJ, Boland PJ, Leung DH, Woodruff JM, Brennan MF (1999). The enigma of desmoid tumors. Ann Surg.

[3] Mankin HJ, Hornicek FJ, Springfield DS (2010). Extra-abdominal desmoid tumors: a report of 234 cases. J Surg Oncol.

[4] Cuomo P, Scoccianti G, Schiavo A, Tortolini V, Wigley C, Muratori F (2021). Extra-abdominal desmoid Tumor fibromatosis: a multicenter EMSOS study. BMC Cancer.

[5] Seinen JM, Niebling MG, Bastiaannet E, Pras B, Hoekstra HJ (2018). Four different treatment strategies in aggressive fibromatosis: a systematic review. Clin Transl Radiat Oncol.

[6] Salas S, Dufresne A, Bui B, Blay J-Y, Terrier P, Ranchere-Vince D (2011). Prognostic factors influencing progression-free survival determined from a series of sporadic desmoid tumors: a wait-and-see policy according to Tumor presentation. J Clin Oncology: Official J Am Soc Clin Oncol.

[7] Fiore M, Rimareix F, Mariani L, Domont J, Collini P, Le Péchoux C (2009). Desmoid-type fibromatosis: a front-line Conservative approach to select patients for surgical treatment. Ann Surg Oncol.

[8] von Mehren M, Kane JM, Bui MM, Choy E, Connelly M, Dry S (2020). NCCN guidelines insights: soft tissue sarcoma, Version 1.2021. J Natl Compr Canc Netw.

[9] Smith K, Desai J, Lazarakis S, Gyorki D (2018). Systematic review of clinical outcomes following various treatment options for patients with Extraabdominal Desmoid tumors. Ann Surg Oncol.

[10] Garcia-Ortega DY, Martín-Tellez KS, Cuellar-Hubbe M, Martínez-Said H, Álvarez-Cano A, Brener-Chaoul M, et al. Desmoid-Type Fibromatosis Cancers (Basel). 2020;12. 10.3390/cancers12071851. 10.3390/cancers12071851 PMC7408653 32660036

[11] Efrima B, Ovadia J, Drukman I, Khoury A, Rath E, Dadia S et al. Cryo-surgery for symptomatic extra-abdominal desmoids. A proof of concept study. J Surg Oncol 2021;124. 10.1002/jso.26528. 10.1002/jso.26528 34043245

[12] Redifer Tremblay K, Lea WB, Neilson JC, King DM, Tutton SM (2019). Percutaneous cryoablation for the treatment of extra-abdominal desmoid tumors. J Surg Oncol.

[13] Kurtz J-E, Buy X, Deschamps F, Sauleau E, Bouhamama A, Toulmonde M (2021). CRYODESMO-O1: a prospective, open phase II study of cryoablation in desmoid tumour patients progressing after medical treatment. Eur J Cancer.

[14] Wang M, Li D, Shang X, Wang J. A review of computer-assisted orthopaedic Surgery systems. Int J Med Rob Comput Assist Surg 2020;16. 10.1002/rcs.2118. 10.1002/rcs.2118 32362063

[15] Hernigou J, Morel X, Hernigou P (2020). Computer Navigation Technique for Simultaneous Total Knee Arthroplasty and opening Wedge High Tibial Osteotomy in patients with large tibial Varus deformity. Surg Technol Int.

[16] McCulloch RA, Frisoni T, Kurunskal V, Maria Donati D, Jeys L. Computer Navigation and 3D Printing in the Surgical Management of Bone Sarcoma. Cells 2021;10. 10.3390/cells10020195. 10.3390/cells10020195 PMC7909290 33498287

[17] Eccles C, Whitaker J, Nyland J, Roberts C, Carlson J, Zamora R. Skin fiducial markers enable accurate computerized navigation resection of simulated soft tissue tumors: a static cadaveric model pilot study. J Surg Oncol. 2018;118. 10.1002/jso.25155. 10.1002/jso.25155 30182459

[18] Lee S, Seong D, Yoon J, Lee S, Baac HW, Lee D, et al. A skin-conformal, stretchable, and breathable fiducial marker patch for surgical navigation systems. Micromachines (Basel). 2020;11. 10.3390/mi11020194. 10.3390/mi11020194 PMC7074652 32070015

[19] Marichal DA, Barnett DW, Meler JD, Layton KF. Fiducial marker placement for intraoperative spine localization. J Vasc Interv Radiol. 2011;22. 10.1016/j.jvir.2010.09.017. 10.1016/j.jvir.2010.09.017 21109457

[20] Imaizumi A, Araki T, Okada H, Sasaki Y, Komiyama T, Suzuki T et al. Transarterial fiducial marker implantation for CyberKnife radiotherapy to treat Pancreatic cancer: an experience with 14 cases. Japanese J Radiol 2021;39. 10.1007/s11604-020-01040-1. 10.1007/s11604-020-01040-1 PMC7813694 32918250

[21] Ayres FJ, Zuffo MK, Rangayyan RM, Boag GS, Filho VO, Valente M (2004). Estimation of the tissue composition of the tumour mass in neuroblastoma using segmented CT images. Med Biol Eng Comput.

[22] Sacks D, McClenny TE, Cardella JF, Lewis CA. Society of Interventional Radiology clinical practice guidelines. J Vasc Interv Radiol. 2003;14. 10.1097/01.rvi.0000094584.83406.3e. :S199-202. 10.1097/01.rvi.0000094584.83406.3e 14514818

[23] Fiore M, MacNeill A, Gronchi A, Colombo C (2016). Desmoid-type fibromatosis: Evolving Treatment standards. Surg Oncol Clin N Am.

[24] Nuyttens JJ, Rust PF, Thomas CRJ, Turrisi AT (2000). Surgery versus radiation therapy for patients with aggressive fibromatosis or desmoid tumors: a comparative review of 22 articles. Cancer.

[25] Vora BMK, Munk PL, Somasundaram N, Ouellette HA, Mallinson PI, Sheikh A, et al. Cryotherapy in extra-abdominal desmoid tumors: a systematic review and metaanalysis. PLoS ONE. 2021;16. 10.1371/journal.pone.0261657. 10.1371/journal.pone.0261657 PMC8699690 34941915

[26] Havez M, Lippa N, Al-Ammari S, Kind M, Stoeckle E, Italiano A (2014). Percutaneous image-guided cryoablation in inoperable extra-abdominal desmoid tumors: a study of tolerability and efficacy. Cardiovasc Intervent Radiol.

[27] Gondim Teixeira PA, Biouichi H, Abou Arab W, Rios M, Sirveaux F, Hossu G (2020). Evidence-based MR imaging follow-up strategy for desmoid-type fibromatosis. Eur Radiol.

[28] Schmitz JJ, Schmit GD, Atwell TD, Callstrom MR, Kurup AN, Weisbrod AJ (2016). Percutaneous cryoablation of Extraabdominal Desmoid tumors: a 10-Year experience. AJR Am J Roentgenol.

[29] Auloge P, Garnon J, Robinson JM, Thenint MA, Koch G, Caudrelier J et al. Percutaneous cryoablation for advanced and refractory extra-abdominal desmoid tumors. Int J Clin Oncol 2021;26. 10.1007/s10147-021-01887-y. 10.1007/s10147-021-01887-y 33709291

[30] Atzmon R, Amar E, Maor D, Rath E (2019). A combined endoscopic and open surgical approach for chronic retracted proximal hamstring avulsion. J Hip Preserv Surg.

